# A critical reflection on the grading of the certainty of evidence in umbrella reviews

**DOI:** 10.1007/s10654-019-00531-4

**Published:** 2019-06-20

**Authors:** Sabrina Schlesinger, Lukas Schwingshackl, Manuela Neuenschwander, Janett Barbaresko

**Affiliations:** 1 Institute for Biometry and Epidemiology, German Diabetes Center, Leibniz Center for Diabetes Research at Heinrich Heine University Düsseldorf, Auf’m Hennekamp 65, 40225 Düsseldorf, Germany; 2 Institute for Evidence in Medicine, Faculty of Medicine and Medical Center, University of Freiburg, Freiburg, Germany

A commentary by Stefania Papatheodorou provided an overview and guidance about umbrella reviews and why they are needed [1]. We would like to compliment the author for providing this short “manual” for conducting an umbrella review.

We read with great interest the commentary, however we have major concerns regarding the grading of the certainty of evidence. Papatheodorou recommended for umbrella reviews to classify the certainty of evidence in four categories (definite association, suggestive (possible) association, no association or inconclusive association (insufficient evidence)) according to the following criteria: the *p* value (statistical significance; *p* < 0.000001), number of cases (cut-off by 1000 cases), the p-value of the largest component study (*p* < 0.05), 95% prediction intervals (excluding the null value), absence of large heterogeneity (I^2^ < 50%), no evidence of small study effects (*p* > 0.1), and no evidence of excessive significance (*p* > 0.1). The author highly recommended to use the criteria to ensure objectivity and standardized classification across umbrella reviews. But so far, there is no consensus that these criteria are the method of choice. Most of these criteria are categorized according to arbitrary cut-offs. For example, the *p*-value is not a good option to evaluate the clinical relevance of the findings from a study [2]. Moreover, using the arbitrary cut-off values for I^2^ has been criticised because it does not present an adequate measure of heterogeneity between studies [3]. Additionally, the 95% prediction interval (PI) is the range in which the true effect size of a future study will lie with 95% certainty. Thus, it provides information on heterogeneity, but should not be misused to interpret an association as “statistical significant” (defined as exclusion of the null value) [3]. We believe that applying these criteria can lead to misclassification of the evidence class. Moreover, we are surprised that the author concluded that “umbrella reviews have the potential to provide the highest quality of evidence, if conducted and interpreted properly”, but did not refer to the study design and quality/risk of bias of the primary studies included in meta-analyses.

In this context, there is a well-established tool available that consider these latter factors. The GRADE (Grading of Recommendations, Assessment, Development and Evaluations) working group has developed a systematic, sensible and transparent approach to grade the certainty of evidence and its strength of recommendations, which are adopted from considerations arising from the Bradford Hill criteria for causation [4]. There is an overlap to the recommended criteria by Papatheodorou, but GRADE implements additional important issues for evaluating the certainty of the evidence. Although GRADE includes some subjective decisions, this tool considers more criteria to evaluate the grading of the evidence [4]. The following criteria are included in the GRADE tool:

- the risk of bias in the studies accounting for limitations in the study design and methods, by applying the Cochrane tools e.g. the revised Cochrane risk-of-bias tool for randomized trials (RoB 2) [5], or the Risk Of Bias In Non-randomized Studies of Interventions (ROBINS-I) [6],
- Inconsistency between study results (heterogeneity),
- Indirectness: Considers the applicability of the single study to the study question of interest with respect to the participants, intervention, comparator and outcome (PICO),
- Imprecision: Precision of the overall effect estimate considering the 95% confidence interval as well as total sample size,
- Small study effect (publication bias),
- Other: Large effect size, dose-response association, absence of confounding.

While the first five categories might be possible reasons for a down grading, the last category may lead to rating up of the certainty of evidence.

The main challenge of the GRADE tool might be the evaluation of risk of bias from the primary studies included in the meta-analysis, by using the tools suggested by Cochrane [5, 6]. So far, especially for non-randomized intervention and observational studies, this information is rarely available in the published meta-analyses. Thus, a lot of work has to be done, which requires a level of epidemiological knowledge of the authors [7].

In conclusion, umbrella reviews provide a comprehensive overview of a specific research topic and are very helpful tools to evaluate the certainty of evidence. These systematic overviews are helpful for the translation of research findings into recommendations, and also for identifying new research directions. We agree that for comparability, standardized procedures, especially regarding the evaluation of the certainty of evidence, are needed. However, the reliance solely on “statistical significance” (expressed mainly as *p*-values) should not be the way forward, and further aspects such as the risk of bias need to be considered to judge the certainty of evidence.

## References

[1] Papatheodorou S (2019). Umbrella reviews: what they are and why we need them. Eur J Epidemiol.

[2] Amrhein V, Greenland S, McShane B (2019). Scientists rise up against statistical significance. Nature.

[3] Borenstein M, Higgins JP, Hedges LV, Rothstein HR (2017). Basics of meta-analysis: I(2) is not an absolute measure of heterogeneity. Res Synth Methods.

[4] Balshem H, Helfand M, Schunemann HJ, Oxman AD, Kunz R, Brozek J, Vist GE, Falck-Ytter Y, Meerpohl J, Norris S, Guyatt GH (2011). GRADE guidelines: 3. Rating the quality of evidence. J Clin Epidemiol.

[5] Higgins JPT, Sterne JAC, Savović J, Page MJ, Hróbjartsson A, Boutron I, Reeve B, Eldridge S. A revised tool for assessing risk of bias in randomized trials. In: Chandler J, McKenzie J, Boutron I, Welch V, editors. Cochrane methods. Cochrane database of systematic reviews 2016;Suppl 1(10): p. 29–31.

[6] Sterne JA, Hernán MA, Reeves BC, Savović J, Berkman ND, Viswanathan M, Henry D, Altman DG, Ansari MT, Boutron I, Carpenter JR, Chan AW, Churchill R, Deeks JJ, Hróbjartsson A, Kirkham J, Jüni P, Loke YK, Pigott TD, Ramsay CR, Regidor D, Rothstein HR, Sandhu L, Santaguida PL, Schünemann HJ, Shea B, Shrier I, Tugwell P, Turner L, Valentine JC, Waddington H, Waters E, Wells GA, Whiting PF, Higgins JP (2016). ROBINS-I: a tool for assessing risk of bias in non-randomised studies of interventions. BMJ.

[7] Thomson H, Craig P, Hilton-Boon M, Campbell M, Katikireddi SV (2018). Applying the ROBINS-I tool to natural experiments: an example from public health. Syst Rev.

